# Bias of Vaccination Coverage in a Household Questionnaire Survey in Japan

**DOI:** 10.2188/jea.15.15

**Published:** 2005-04-22

**Authors:** Shuji Hashimoto, Miyuki Kawado, Rumi Seko, Masahiro Kato, Nobuhiko Okabe

**Affiliations:** 1 Department of Hygiene, Fujita Health University School of Medicine.; 2 Faculty of Nursing, Fujita Health University School of Health Sciences.; 3 Chita Public Health Center, Aichi Prefecture.; 4 Infectious Disease Surveillance Center, National Institute of Infectious Diseases.

**Keywords:** vaccination coverage, household survey, polio, measles, bias

## Abstract

BACKGROUND: Although a household questionnaire survey is important for estimating vaccination coverage, it raises several problematic issues.

METHODS: A household survey was conducted on 900 subjects aged 2, 4, and 6 years living in Obu City, Japan, and a second survey for non-respondents to the first survey was then conducted. Questionnaires bearing a subject’s name were used for half of the subjects, while the others were anonymous (the named and nameless groups, respectively). The vaccination dates of six kinds of vaccines, including poliovirus and measles vaccine, for those in the named group were reviewed using the administrative records at the Obu City Health Center.

RESULTS: The response rate was 70.1% in the first survey and 84.1% in the first and second surveys combined. The response rate for both groups was nearly equal. Based on administrative records in the named group, the vaccination coverage in the respondents was 0.9-2.9% higher than that in total subjects, and that in the respondents to the first survey was 0.8-4.9% higher. There were very few inconsistencies in the vaccination status between responses to the questionnaire and data of administrative records among respondents in the named group.

CONCLUSIONS: These results suggested that vaccination coverage from a household questionnaire survey in Japan might not be extremely biased by either non-responses or incorrect answers.

National vaccination programs for infectious diseases, including polio and measles, have been conducted in most countries around the world.^1^^,^^2^ Sufficiently complete vaccination coverage is important for preventing and controlling epidemics of such infectious diseases.^2^^,^^3^

The data of vaccinations performed by service providers (administrative records) are available for roughly estimating vaccination coverage in a target population (e.g., population aged 1-3 years), but are not useful in providing accurate estimates unless they include the age-specific number of persons vaccinated each year.^4^^,^^5^ Household questionnaire surveys on individual vaccination histories are another source used in many countries.^3^ Although such surveys directly provide the data of vaccination coverage in a target population, they raise several problematic issues.^6^ Non-responses and incorrect answers tend to induce biases in vaccination coverage.^7^^,^^8^

In Japan, as well as in many other countries, national statistics from administrative records include no information on the age of vaccinated persons.^9^ When planning a national household questionnaire survey, taking account of such a problematic issue is important.

In the present study, a household questionnaire survey of vaccinations in Obu City, Japan was conducted in collaboration with the Obu City Health Center. Based on the resultant data, the biases in vaccination coverage due to non-responses and incorrect answers were evaluated.

## METHODS

### Vaccination in Japan

BCG vaccination for negative cases of a tuberculin test among those aged 4 years or younger is strongly recommended by the Tuberculosis Preventive Law of Japan in 2003.^10^^,^^11^ Six kinds of vaccinations for children aged 7 years or younger are also strongly recommended by Japan’s Immunization Law, i.e., diphtheria and tetanus toxoids, acellular pertussis vaccine (DTP), poliovirus, measles, rubella and Japanese encephalitis vaccines. When a child is vaccinated, the vaccination date is recorded in his/her maternal and child health handbook.

### Subjects

Subjects were randomly selected from all children aged 2, 4 and 6 years living in Obu City as of December 2003 using the population registries. The number of subjects was 300 in each age group for a total of 900. Table 1 shows the population size and the number of subjects.

**Table 1.** Population size and the number of subjects.

Age group	Population size	No. of subjects	(%)
2 years old	880	300	34.1
4 years old	824	300	36.4
6 years old	767	300	39.1

Total	2,471	900	36.4

### Data collection

The first survey was conducted by mail in December 2003. Half of the subjects were randomly allocated, and a questionnaire bearing their name was sent to their parents together with a written request for a response from the research group and the Obu City Health Center (the named group). For the others, we used a questionnaire without their name together with a postcard bearing their name, which they were asked to send separately if they submitted a completed questionnaire (the nameless group). For subjects whose named questionnaire or named postcard was not returned, a second survey was conducted in January 2004 using the same methods. The questionnaires with and without the subject’s name were used in the named and nameless group, respectively.

The questionnaires sent to both the named and nameless groups covered sex, date of birth, and vaccination dates of BCG and the six kinds of vaccines mentioned above. They were asked to confirm their vaccination date by referring to each subject’s maternal and child health handbook. Responses to the vaccination dates of BCG and the six kinds of vaccines submitted by those in the named group were checked against the administrative records at the Obu City Health Center in March 2004 based on the personal identifiers (name, address, sex, and date of birth).

### Data analysis

Response rates of the first and second surveys were compared between the named and nameless groups. For the named group, vaccination coverage as of December 1, 2003 based on the administrative records was compared among respondents to the first survey (first respondents), those to the second survey (second respondents), and non-respondents using the chi-squared test. Data on the vaccination status as of December 1, 2003 based on responses to the questionnaire in the named group were compared with the corresponding data in the administrative records.

In the above two analyses of the vaccinations in the named group, subjects whose BCG vaccination was not recorded on the administrative records were excluded, and the six kinds of vaccines (except BCG) were examined, because a vaccination prior to moving to Obu City might not be recorded on the administrative records at the Obu City Health Center and because BCG vaccination is usually earliest among those vaccines.

### Ethics approval

This study was approved on November 2003 by the Ethical Board of the Fujita Health University School of Medicine.

## RESULTS

Table 2 shows the total number of respondents in the named and nameless groups, i.e., 631 (70.1%) in the first survey, 126 (14.0%) in the second survey, and 757 (84.1%) in both surveys. The response rate for both groups was nearly equal. The number of postcards returned by the nameless group was 377, which was equal to the number of the returned questionnaires.

**Table 2.** The number of respondents in the named and nameless groups.

	Named group	(%)	Nameless group	(%)	Total	(%)
No. of subjects	450	(100)	450	(100)	900	(100)

No. of first respondents	319	(70.9)	312	(69.3)	631	(70.1)

No. of second respondents	61	(13.6)	65	(14.4)	126	(14.0)

No. of respondents	380	(84.4)	377	(83.8)	757	(84.1)

Table 3 shows vaccination coverage in the 388 subjects of the named group based on administrative records. The vaccination coverage in subjects ranged between 71.6 and 97.4% for the first, second, third and additional doses of DTP, 87.1-95.1% for the first and second doses of poliovirus, 94.8% for measles vaccine, 88.4% for rubella vaccine, and 30.9-54.4% for the first, second and additional doses of Japanese encephalitis vaccine. The vaccination coverage in first respondents was higher than that in second respondents, while that in non-respondents was lowest. A significant (p<0.05) difference among these groups was observed in the first, second, third and additional doses of DTP, the second dose of poliovirus, rubella vaccine and the additional dose of Japanese encephalitis vaccine. The difference in vaccination coverage between all respondents and non-respondents ranged between 6.1 and 19.3% among the six kinds of vaccines. The vaccination coverage in all respondents ranged between 0.9 and 2.9% higher than that in total subjects, and that in first respondents ranged between 0.8 and 4.9% higher.

**Table 3.** Vaccination coverage in the named group based on administrative records.

		First respondents		Second respondents		Non- respondents		Subjects			Difference		

											All respondents vs. non- respondents	All respondents vs. subjects	First respondents vs. subjects
			
		No.	(%)	No.	(%)	No.	(%)	No.	(%)		(%)	(%)	(%)
No. of persons		273	100	57	100	58	100	388	100				

DTP^†^	First dose	270	98.9	55	96.5	53	91.4	378	97.4	*	7.1	1.1	1.5
	Second dose	269	98.5	54	94.7	51	87.9	374	96.4	**	9.9	1.5	2.1
	Third dose	267	97.8	52	91.2	47	81.0	366	94.3	**	15.6	2.3	3.5
	Additional dose	209	76.6	34	59.6	35	60.3	278	71.6	**	13.3	2.0	4.9

Poliovirus	First dose	262	96.0	55	96.5	52	89.7	369	95.1		6.4	1.0	0.9
	Second dose	251	91.9	46	80.7	41	70.7	338	87.1	**	19.3	2.9	4.8

Measles vaccine		261	95.6	55	96.5	52	89.7	368	94.8		6.1	0.9	0.8

Rubella vaccine		249	91.2	47	82.5	47	81.0	343	88.4	*	8.7	1.3	2.8

Japanese encephalitis vaccine	First dose	154	56.4	30	52.6	27	46.6	211	54.4		9.2	1.4	2.0
	Second dose	149	54.6	29	50.9	23	39.7	201	51.8		14.3	2.1	2.8
	Additional dose	94	34.4	16	28.1	10	17.2	120	30.9	*	16.1	2.4	3.5

In this analysis, the data of 388 subjects whose BCG vaccination was entered in administractive records were used.

* p<0.05, ** p<0.01 for chi-squared test.

† : diphtheria and tetanus toxoids, and acellular pertussis vaccine.

Table 4 shows the vaccination status based on the responses to the questionnaire and data in the administrative records for 330 respondents of the named group. The inconsistencies in vaccination status (vaccinated vs. not vaccinated or vice versa) between the responses and the administrative records occurred in 0-5 of respondents.

**Table 4.** Vaccination status in responses to questionnaires and in administrative record data among respondents of the named group.

Questionnaire:		Administrative records					
		Vaccinated			Not vaccinated		
	
		Vaccinated	Not vaccinated	Unknown	Vaccinated	Not vaccinated	Unknown
DTP^†^	First dose	322	2	1	0	4	1
	Second dose	320	2	1	1	6	0
	Third dose	315	3	1	1	10	0
	Additional dose	240	2	1	3	84	0

Poliovirus	First dose	316	0	1	3	10	0
	Second dose	295	1	1	3	30	0

Measles vaccine		314	1	1	2	12	0

Rubella vaccine		293	1	2	1	33	0

Japanese encephalitis vaccine	First dose	183	0	1	0	146	0
	Second dose	177	0	1	1	151	0
	Additional dose	110	0	0	0	219	1

In this analysis, the data of 330 respondents whose BCG vaccination was entered in administrative records were used.

† : diphtheria and tetanus toxoids, and acellular pertussis vaccine.

In the above two analyses of the vaccinations in the named group, 62 subjects whose BCG vaccination was not recorded in the administrative records were excluded. Those included 50 respondents (81%) in the first and second surveys combined. Among their responses to the BCG vaccination question, 46 (92%) were vaccinated, 2 (4%) were not vaccinated, and 2 (4%) were unknown.

## DISCUSSION

In general, a nameless questionnaire is useful for household surveys of vaccinations. In this study, to compare the responses to the questionnaire with data in the administrative records, both the named and nameless ones were used. The response rate of 84% was identical in both groups. The use of a written request for a response from both the research group and the Obu City Health Center probably contributed to the high response rates, as did the second survey for non-respondents to the first survey. If that is so, this finding suggests the feasibility of using either the named or nameless questionnaire in a national household survey of vaccinations in Japan.

The vaccination coverage in non-respondents of the named group was lower than that in respondents. Several previous studies reported the characteristics of non-respondents to questionnaires on smoking,^12^^-^^14^ but few studies concerning vaccination indicated results similar to ours.^7^

The vaccination coverage in total subjects in the present study was overestimated based on the data of respondents. The bias of vaccination coverage was less than 3% when estimated using the data of all respondents, and less than 5% when using the data of first respondents only. The response rate was 85% in the former case and 70% in the latter. When planning a national household questionnaire survey on the vaccination coverage in Japan, taking account of the precision and bias of the estimate would be important. Although the population and method in the national survey might be different from those in our survey, results of the response rate and the bias in vaccination coverage due to non-responses would prove helpful.

Discrepancies in vaccination status between the responses to the questionnaire and the data in administrative records among respondents of the named group were rare. Several previous studies reported that vaccination status was biased by parent’s recall.^8^^,^^15^ One reason for the rarity of discrepancies in our survey would be that the vaccination dates in the responses to the questionnaire were confirmed to be the same dates recorded in the maternal and child health handbook. This finding suggested that the vaccination coverage obtained from a household questionnaire survey in Japan might not be greatly biased by incorrect answers if the subjects were asked to confirm the vaccination date by referring to their own maternal and child health handbook.

There are several limitations and problems in the present study. The bias of the vaccination coverage due to non-responses in our household survey was evaluated for the named group, but not for total subjects. The bias due to incorrect answers was analyzed for respondents, but not for non-respondents. Since the administrative records were incomplete, 62 subjects whose BCG vaccination was not recorded in the administrative records were excluded in our analyses. If they are randomly selected from total subjects, their response rate to the questionnaire and their vaccination coverage are expected to be equal to those in total subjects, and the bias in the vaccination coverage due to non-responses is accurately estimated using the data of subjects without them. In our survey, their response rate of 81% was slightly lower than 85% in the others. Among their responses to the BCG vaccination question, 46 (92%) were vaccinated, 2 (4%) were not vaccinated, and 2 (4%) were unknown. Thus, their selection would not be random, and the bias in the vaccination coverage due to non-responses by our analysis might not be accurately estimated. Although we conducted this survey only and our analyses had several limitations and the problems mentioned above, our findings would provide important information for planning a national household questionnaire survey on the vaccination coverage in Japan.
